# Study on Manufacturing via Slip Casting and Properties of Alumina-Titanium Composite Enhanced by Thialite Phase

**DOI:** 10.3390/ma16010079

**Published:** 2022-12-22

**Authors:** Marcin Wachowski, Justyna Zygmuntowicz, Robert Kosturek, Paulina Piotrkiewicz, Radosław Żurowski, Anna Więcław-Midor, Lucjan Śnieżek

**Affiliations:** 1 Faculty of Mechanical Engineering, Military University of Technology, 2 gen. S. Kaliskiego Str., 00-908 Warsaw, Poland; 2 Faculty of Materials Science and Engineering, Warsaw University of Technology, 141 Woloska Str., 02-507 Warsaw, Poland; 3 Faculty of Chemistry, Warsaw University of Technology, 3 Noakowskiego Str., 00-664 Warsaw, Poland

**Keywords:** thermogravimetric, slip casting, Al_2_O_3_/Ti system, thialite

## Abstract

This paper aims to study the Al_2_O_3_/Ti ceramic-metal composite obtained by the slip casting method. Samples containing 50% volume of the solid phase, including 10% volume of the metallic phase, were investigated. The rheological properties were analyzed. Thermogravimetric analysis was performed. The properties of the obtained composite determined the phase composition using and SEM/EDS microstructural analysis and the XRD method. The size of the titanium particles equals 20.6 ± 10.1 mm, which corresponds to 27.5% of the initial size and indicates significant fragmentation of the titanium powder during the manufacturing of the composite. The relative density of the fabricated composites was equal to 99%. The slip casting method allows to obtain the proposed composite additionally enhanced by the presence of TiO_2_ and Al_2_TiO_5_ (thialite). Research results revealed a non-Newtonian character of the composite suspension flow with clear thinning under the influence of increasing shear forces. The obtained composites are characterized by the lack of visible defects (cracks, microcracks and delamination) on the surface.

## 1. Introduction

Machine elements operating in extreme conditions, such as engine valves, thermal barriers or turbine blades, are often exposed to the simultaneous occurrence of a number of adverse external factors, including intense abrasion or erosion, high temperature, aggressive chemical environment and high impact loads. Due to the high number of requirements for construction materials used for these types of components, the choice of material is quite narrow. Among the groups of qualifying materials that meet these requirements, the increasingly popular technical ceramics and composites on which they are based deserve special attention. It is not without reason that a significant number of the components for the automotive industry designated to work in extreme conditions is currently made of Si_3_N_4_ [1,2], and that SiC/SiC ceramic matrix composite are displacing nickel superalloys as a material for high-pressure turbine blades [3]. Considering the need to transfer significant impact loads, one of the leading roles among modern technical ceramics is played by Al_2_O_3_ [4] and its composites [5,6,7,8]. The introduction of additional particles, most often metallic, into the Al_2_O_3_ matrix, allows significantly increasing the strength of the composite obtained in this way, especially in terms of fracture toughness [6,7,9]. The decisive role here is played by the ability of the metallic phase to dissipate energy by plastic deformation, as well as blocking and bridging cracks [9]. In the literature reports, among the metallic particles introduced into Al_2_O_3_, one can find, among others, aluminum [10], copper [11], titanium [12,13,14,15], nickel [16,17], and iron [18].

Due to favorable mechanical properties and small differences in density between the matrix and the reinforcement, Al_2_O_3_-Ti composites attract considerable attention in the development of modern technology. Meir et al. investigated the mechanical properties of Al_2_O_3_-Ti composites produced by spark plasma sintering (SPS) [12]. With 20% titanium, he found an increase in flexural strength to the value of 767 MPa. The basic mechanism of decohesion of the composites produced in this way was the cracking of the Al_2_O_3_ matrix. A similar study by Horvitz et al. focused on obtaining the Al_2_O_3_-Ti composite using self-propagating high-temperature synthesis (SHS) [13]. As a result of the reaction of titanium dioxide and aluminum, the target composite was obtained with the additional participation of fine intermetallic TiAl and Ti_3_Al particles, evenly distributed in the matrix. Another approach to the production of Al_2_O_3_-Ti composites is proposed by Edalati et al.: high-pressure torsion (HPT) and subsequent annealing [19]. The resulting composite with 18% titanium content was characterized by a high microhardness value of approx. 650 HV, which the author associated with the formation of the Ti_3_Al intermetallic phase and the formation of a solid solution of Al and O in Ti formed by diffusion along grain boundaries. Most of the literature reports indicate the SPS technique as an effective method for the production of Al_2_O_3_-Ti composites [12,20,21]. An important issue in the sintering of these composites is the need to limit the formation of intermetallic compounds and solid solutions at the Ti/Al_2_O_3_ interface, leading to a weakening of grain boundary cohesion and, as a result, the deterioration of mechanical properties [12,22,23].

A significant knowledge gap is posed by the properties of Al_2_O_3_-Ti composites produced by other techniques. In this work, the authors propose the use of a two-stage process consisting of slip casting and sintering for the production of the discussed composites, which has already been successfully used to produce other Al_2_O_3_-metal composites [16,24,25,26]. The goal was to produce a composite with a metallic phase share of 10%, which would give the best properties in terms of strength [12,19].

The investigated composite, characterized by high strength parameters by a strengthening phase, can be dedicated to special application works at high temperatures and in aggressive environments, i.e., production processes in chemical reactors. The applied material must exhibit adequate chemical resistance, resistance to high temperatures and abrasion resistance. At the same time, a high level of mechanical properties at elevated temperatures is required, especially resistance to cracking. 

The paper reveals the results of the interdisciplinary investigation, combining the fields of materials engineering and colloid chemistry. This work focuses on the production and characterization of composite samples from the Al_2_O_3_-Ti system. Samples containing 50% volume of the solid phase, including 10% volume of the metallic phase, were produced by slip casting. The determination of rheological properties is essential in the preparation of ceramic suspensions or pastes used in methods for the preparation of ceramic-metal materials. Therefore, in the first step, the rheological properties of ceramic slips based on Al_2_O_3_ and Ti were determined. A thermogravimetric analysis was carried out, which made it possible to determine the temperatures at which complete thermal decomposition of the organic additives used in the slips used for forming the samples takes place. The thermogravimetric properties of ceramic slips constitute a scientific gap in current knowledge, despite the fact that this is an important issue in terms of controlling the purity of an obtained composite. Next, the density of sinters was examined using the pycnometric method. To characterize the phase structure of the molds before and after the sintering process, XRD analysis was carried out. The microstructure of the samples was identified based on a scanning electron microscope observation. The obtained investigation results make a significant contribution to the development of fundamental knowledge in the field of ceramic-metal composites.

## 2. Materials and Methods

### 2.1. Materials Used for Research

The base materials were Al_2_O_3_ powder (TM-DAR trade name) from the Tamei Chemicals Co. (Tokyo, Japan) and titanium powder (GoodFellow Cambridge Limited, Huntingdon, UK). Powder specifications provided by manufacturers are shown in Table 1.

### 2.2. Sample Preparation Process

Composite samples from the Al_2_O_3_/Ti system were made using the slip casting method. The process of producing Al_2_O_3_/Ti composites was performed by the following procedure:

- Weigh the right amounts of powders, fluidizer and water;
- Combine and mix the weighed ingredients to obtain a homogenous suspension.

In the first stage, weighed fluidizers in the form of citric acid and diammonium hydrogen citrate were dissolved in distilled water. Citric acid was applied in an amount of 0.1 wt.%, while DAC was applied in an amount of 0.3 wt.%, both with respect to the total weight of the powders. The key role of the fluidizers in the process involves providing stability to the slurry from the moment it is made until the casting, ensuring that sedimentation does not occur. In the next step, the previously weighed powders were poured into a mixture of water and fluidizer in order to thoroughly combine the ingredients. The composition of the slip used to form the samples by slip casting was selected on the basis of experimental studies. A slip from the Al_2_O_3_/Ti system containing 50% volume of the solid phase was prepared, including 10% volume of the metallic phase.

- Procedure of homogenization and deaeration

Homogenization was carried out in two stages [27,28]. Firstly, the suspension was homogenized in the Retsch PM 400 planetary-ball mill with 4 grinding stations. The process was performed for 1 h with 300 rpm rotational speed. During homogenization, vessels with suspensions are placed eccentrically on the rotating base of the planetary-ball mill. The rotation direction of the mill base is opposite to the direction of rotation of the vessels. The speed ratio was 1:2 during the investigation. The 8:1 ball-to-powder weight ratio was employed. Balls with a diameter of 10 and 12 mm were utilized. Then, in the second stage, the prepared slurry was placed in a high-speed homogenizer (THINKY ARE-250 model, Thinky, Tokyo, Japan). The first stage of homogenization in the homogenizer device was mixing for 8 min at a 1000 rpm rotational speed. Next, deaeration of the mixture for 2 min at a 2000 rpm rotational speed was applied.

- Casting of the deaerated mixture was performed in a previously prepared cylindrical mold made of gypsum. The diameter of the mold was 20 mm and the height was 10 mm.

The use of gypsum molds allows the liquid medium to be removed from the prepared slip. The molds were prepared from a mixture of gypsum and water in a ratio of 4:3. As a result of the action of capillary forces, the liquid medium was drained from the fitting, resulting in a permanent representation of the form. The shape obtained in this way is called “green body”; it reproduces the shape and is durable enough to be subjected to the forming process, i.e., grinding, drying and sintering.

- Sample drying process

The samples were dried in a laboratory at 40 °C for 48 h.

- Mechanical treatment

The samples were prepared by grinding on sandpaper with the following gradations: 120, 400, 600, 800 and 1200 to obtain a profile with flat-parallel surfaces.

- Sintering of samples

The sintering process was performed in a Carbolite chamber furnace (Carbolite·Gero, Neuhausen, Germany) of the HTF 17/5 type, model 21-100324, 220 Volt, 50–60 Hz, 19,5 A, 4050 Watt. The samples were heated 5 °C/min from the temperature of 25 °C → 1450 °C, then the fittings were kept for 2 h at a temperature of 1450 °C, and then the cooling process took place at the rate of 5 °C/min from the temperature of 1450 °C → 25 °C. The sintering process was carried out in an aerated atmosphere. 

### 2.3. Research Methods

To confirm the density of the powders specified by the manufacturer, the density was measured using an AccuPyc II 1340 helium pycnometer from Micrometrics (Norcross, GA, USA), using a cylindrical measuring chamber with a volume of 2.7814 [cm^3^]. Measurement chamber filling pressure with helium: 19.5 psig., measurement accuracy: 0.03% and measurement repeatability: ±0.01%. The powders were investigated in the helium at the parameters of 700 cycles and 10 rinses. 

XRD studies were carried out to characterize the phases of the starting powders and shapes in the raw and sintered states. Measurements were performed using the Rigaku Mini Flex II diffractogram (Rigaku, Tokyo, Japan) with CuKα radiation and wavelength equal λ = 1.54178 Å. Angular range 2θ—20–100°, voltage—30 kV, current—15 mA, measurement step—0.01° and counting time—1 s were applied.

The slurry sedimentation test was carried out to confirm the stability of the slurry produced during the technological process. The stability of the suspension was analyzed by carrying out its macroscopic observations as a function of time from the moment of preparation. During the observations, containers with suspensions were photographed at specified time intervals from the moment of casting for 1 h to determine the tendency to sedimentation. The prepared suspension was poured into a glass, sealed container with a volume of 10 cm^3^. The glass container with the slip was placed on a previously prepared measuring station and subjected to macroscopic observations for the occurrence of sedimentation. Pictures documenting the state of the prepared suspension were taken every 3 min from the start of the observation (the starting point was set as 0 min) for a period of 36 min.

The rheological properties of the slip were tested using a Kinexus Pro rotational rheometer from Malvern Instruments (Malvern, Great Britain). The tests were carried out in a plate-plate system with a 0.5 mm gap. Experiments were performed for increasing and then decreasing shear rates in the range of 0.1 s^−1^ to 100 s^−1^ and from 100 s^−1^ to 0.1 s^−1^.

Thermogravimetric tests were performed using the STA 449C device (Netzsch, Selb, Germany) coupled with the QMS 403C mass spectrometer (Netzsch, Selb, Germany). The measurements were carried out in an atmosphere of synthetic air, i.e., a mixture of nitrogen and oxygen in a volume ratio of 79:21. The samples were heated at a constant rate of 5 °C/min to 1450 °C. The mass spectrometer recorded the mass-to-charge ratio (*m*/*z*) of ionized particles in the range of 10–300, as well as their intensity during thermogravimetric measurements.

Microstructural observations of the base powders and sintered composites were carried out using a scanning electron microscope (SEM), (JEOL, Tokyo, Japan) of the JEOL JSM-6610 type with the use of a secondary electron detector (SE). Applied accelerating voltage was 15 kV. Due to the low conductivity of the material, additional metallization of the sample was necessary. For metallization of the samples, carbon target was applied. To determine the chemical composition of the fabricated composite, surface microanalyses were performed using an Oxford X-Max electro-dispersive spectrometer (EDS), (Oxford Instruments, Oxfordshire, UK).

## 3. Results and Discussion

In the first stage of the research, the true density of the aluminum oxide and titanium powder was determined using the pycnometric method. The obtained results are presented in Table 2. The tests showed that the determined real densities of Al_2_O_3_ and Ti powders are the same as the theoretical density declared by the manufacturer. In addition, in order to confirm the purity of the powder used in the experiment, an XRD test was carried out. The phase analysis of the Al_2_O_3_ powder was carried out in order to exclude phases other than the α-Al_2_O_3_ phase declared by the manufacturer.

**Figure 1:**
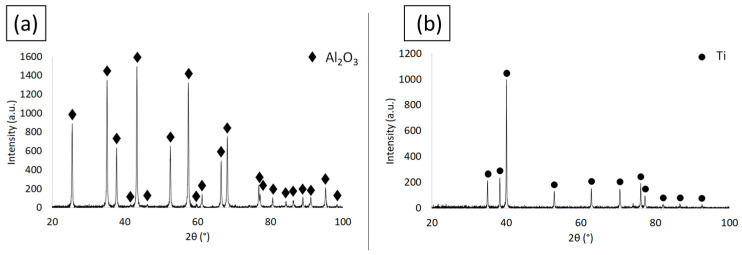
Diffractograms of the starting powders: (**a**) Al_2_O_3_, (**b**) Ti.

Figure 1 shows the results of the phase analysis for alumina powder (Figure 1a) and titanium (Figure 1b). The analysis of the obtained results for alumina was carried out on the basis of safety data sheets with the number PDF #04-003-7263 and for titanium on the basis of PDF data sheet #01-083-5019. Analysis of the diffraction pattern revealed that there are no peaks from other phases, which confirms the high purity of the powders declared by the manufacturers. 

**Figure 2:**
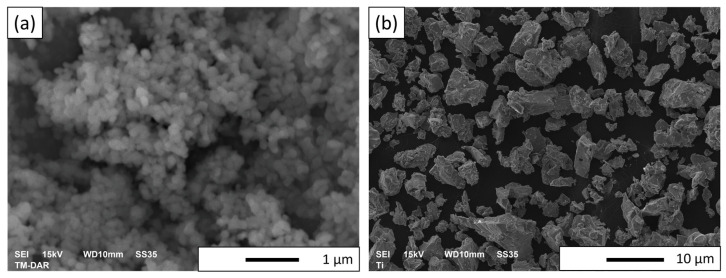
Sample SEM pictures of Al_2_O_3_ (**a**) and Ti (**b**) powder.

Microstructural observations of the Al_2_O_3_ powder were performed as well. Figure 2 shows example SEM images of alumina (a) and titanium (b) powders. Research results revealed that the alumina powder is distinguished by its tendency to form agglomerates. In addition, by analyzing the microphotographs, it can be seen that the Al_2_O_3_ powder is characterized by grains of slightly different shapes. It was found that the alumina powder particles have a coaxial shape with a tendency toward agglomeration, while the titanium powder particles have a flake shape.

**Figure 3:**
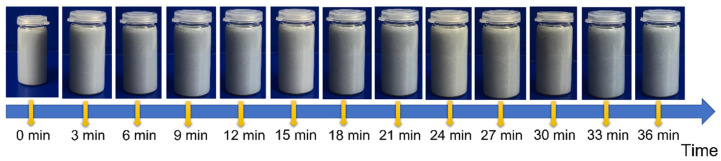
Sedimentation test of the slip from the Al_2_O_3_/Ti system containing 50% volume of the solid phase, including 10% volume of the metallic phase.

In order to efficiently form samples using the slip casting method, as well as to produce molds with appropriate properties, the suspension used must be properly dispersed while maintaining stability over time. The purpose of the sedimentation test was to check whether the slip used in the experiment was stable over time. Figure 3 shows a combination of photos documenting the state of suspension at specific time intervals. Based on macroscopic observations, no changes in the appearance of the prepared suspension in a transparent vessel were found. Observations of the slip from the Al_2_O_3_/Ti system containing 50% volume of the solid phase were performed, including 10% volume of the metallic phase, proving it to be stable throughout the test.

**Figure 4:**
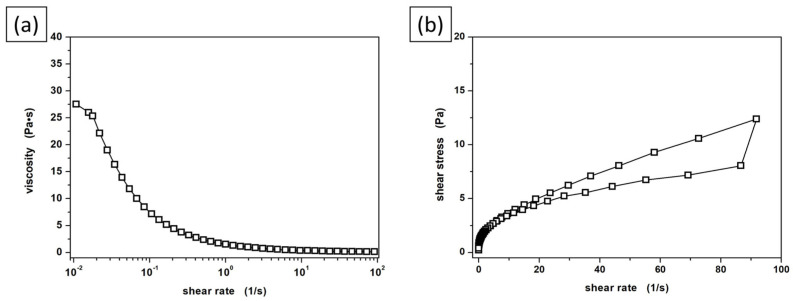
Viscosity curve (**a**) and flow curve (**b**) of the prepared ceramic slurry.

In the next stage of the research, the basic rheological properties of the used ceramic-metallic suspension were determined. Figure 4a presents its viscosity curve. The suspension exhibits a non-Newtonian character of flow with clear thinning under the influence of increasing shear forces. The reason for the drop in suspension viscosity is obviously the decreasing internal resistance during increasing stress, due to the parallel arrangement of fluid components in relation to the direction of the flow. The reduction of the dynamic viscosity value in the tested shear rate range is presented in Table 3. According to our earlier reports, such a viscosity curve profile is typical for aqueous ceramic suspensions [16,29,30,31]. It is also worth noting that the ceramic suspension used in the research is characterized by thixotropic properties, as evidenced by the presence of hysteresis loops in the flow curve (Figure 4b).

**Figure 5:**
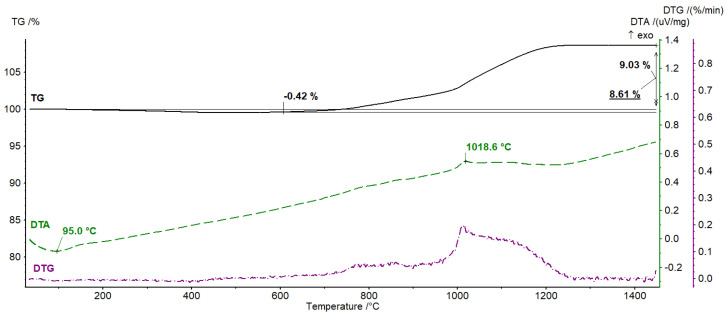
Thermal analysis results—DTA, TG and DTG curves.

Then, in order to determine the changes occurring during the sintering process, thermogravimetric analysis was carried out. Figure 5 shows the results of the thermal analysis-DTA, TG and DTG curves for the Al_2_O_3_/Ti sample containing 50% vol. of the solid phase, including 10% vol. of the metallic phase. The TG curve shows a two-stage change in the mass of the tested sample. In the first step, up to a temperature of approx. 600 °C, a weight loss of 0.43% is observed, which is associated with the dehydration process and the decomposition of organic additives, such as fluidizers, which were used during the preparation of the ceramic mass. In the second stage, a significant (9.03%) increase in mass is visible in the temperature range of 600–1250 °C. In this temperature range, we also observe a peak on the DTG curve, which proves the occurrence of intensive changes in this range. The endothermic peak on the DTA curve with the minimum at 95 °C is visible. This peak overlaps with the peak from the mass spectrometer of *m*/*z* value 18 (109.1 °C) which can be ascribed to H_2_O; therefore, it indicates the dehydration process. The DTA curve also shows an exothermic peak with a maximum at 1018.6 °C, which indicates the thermal nature of the processes taking place at this stage. The increase in mass probably results from the transformations of Ti and TiO_2_ resulting from their oxidation, as well as from the formation of compounds that include Al_2_O_3_, TiO_2_ and Al_2_TiO_5_.

**Figure 6:**
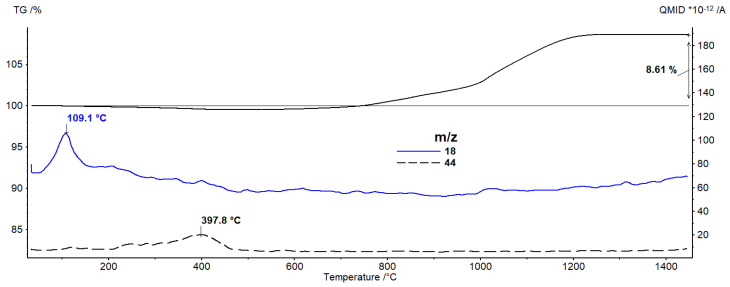
The results of thermal analysis showing changes in the intensity of the m/z value as a function of temperature for the Al_2_O_3_/Ti sample.

Then, Figure 6 presents the results of thermal analysis showing changes in the intensity of the *m*/*z* (mass to charge ratio) value as a function of temperature for the Al_2_O_3_/Ti sample containing 50% vol. of the solid phase, including 10% vol. of the metallic phase. For the test sample, two signals from the mass spectrometer with *m*/*z* values of 18 and 44 were recorded, which can be attributed to water and CO_2_, respectively. Both curves show peaks with maxima of 109.1 °C for the *m*/*z* 18 curve and 397.8 °C for the *m*/*z* 44 curve. They arise below the temperature of 600 °C, which confirms the mass loss observed on the TG curve is related to the distribution of individual components of the mass from which the test sample was made (water, citric acid and diammonium citrate—DAC). Such a small weight loss is puzzling, considering that the percentage of water and organic additives is about 20%.

Macroscopic observations of the molds after the sintering process revealed that the composites are characterized by a lack of surface defects such as cracks, microcracks and delamination. In the next step, the density of the samples before and after the sintering process was determined using the pycnometric method. The raw samples had a density of 4.02 g/cm^3^, while after the sintering process the density was 3.89 g/cm^3^. The relative density of the molds obtained was 99%. 

**Figure 7:**
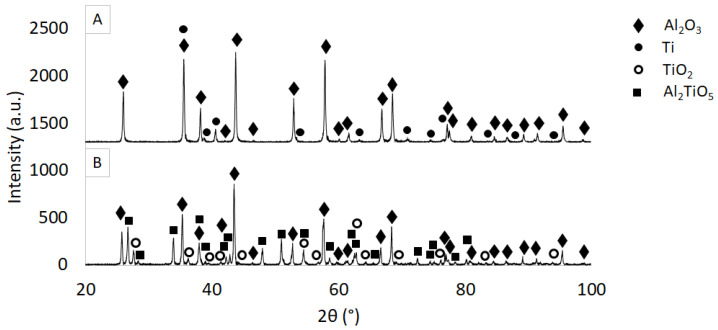
Diffractogram for a selected Al_2_O_3_/Ti sample in the raw state (**A**) and after sintering (**B**).

Then, XRD analysis was carried out for the obtained samples in the raw state and after the sintering process. Figure 7 shows the combined diffraction pattern for raw and sintered samples with phases matched to individual peaks. XRD analysis of the raw samples confirmed the presence of two phases in the composite: hexagonal Al_2_O_3_ (#04-013-1687) and hexagonal Ti (#00-044-1294). Using direct measurements, the characteristic spectra of the composite sample before the sintering process were determined. From these, it was found that the observed reflections for Ti phase at 2θ angle were, respectively: 35.52°, 38.74°, 40.54°, 53.32°, 63.25°, 70.88°, 74.55°, 76.68°, 82.40°, 86.92° and 92.87°, which originate from the following families of crystallographic planes, respectively: (100), (002), (101), (102), (110), (103), (200), (112), (004), (202) and (104).

However, in sintered composites, it was found that the samples are characterized by the presence of three phases: Al_2_O_3_, TiO_2_, Al_2_TiO_5_. Analysis of the phase composition of the sintered samples, in addition to Al_2_O_3_ (#04-013-1687), revealed the presence of new phases in the composite: tetragonal TiO_2_ (#03-065-1118) and orthorhombic Al_2_TiO_5_ (#00-041-025). Titanium was not detected in the sintered samples. Analysis of the spectrum obtained made it possible to isolate the characteristic reflections for each phase and to determine from which plane families they originate. For TiO_2_, 13 reflections with the highest intensity originating from the following families of planes were identified in the spectrum: (110), (101), (200), (111), (210), (211), (220), (002), (310), (112), (202), (321) and (411). Corresponding reflections were observed in the diffractogram at the following values of 2θ angle equal to: 27.58°, 36.18°, 39.36°, 41.30°, 44.16°, 54.46°, 56.73°, 62.78°, 64.30°, 69.97°, 76.96°, 82.08° and 93.85°. The analysis of the reflections from Al_2_TiO_5_, on the other hand, allowed the 19 reflections with the highest intensity to be determined on the diffractogram obtained. These were observed at the following values of 2θ angle: 26.68°, 28.32°, 33.90°, 37.94°, 38.94°, 42.22°, 42.80°, 47.94°, 50.94°, 54.46°, 58.54°, 62.48°, 62.78°, 65.43°, 72.44°, 74.48°, 75.09°, 78.38° and 80.23°. The listed reflections correspond to crystallographic planes: (101), (111), (230), (301), (131), (240), (420), (430), (002), (341), (521), (232), (531), (402), (432), (171), (252), (721) and (062).

An air atmosphere enabled the reaction between titanium and oxygen to occur, resulting in titanium (IV) oxide formation. The reaction of TiO_2_ formation leads spontaneously towards the formation of oxides, since the free energy ΔG has a negative value. In the further stage of sintering, the formed titanium (IV) oxygen can react with aluminum oxide, resulting in the formation of Al_2_TiO_5_ (thialite) and TiO_2_/Al_2_O_3_ phases [32,33,34].

**Figure 8:**
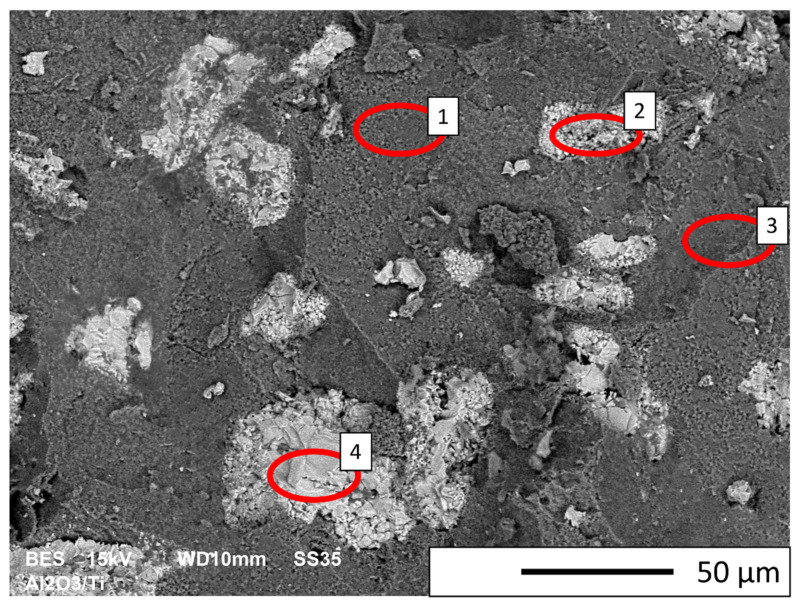
SEM image (BSE mode) of sintered Al_2_O_3_/Ti sample.

**Figure 9:**
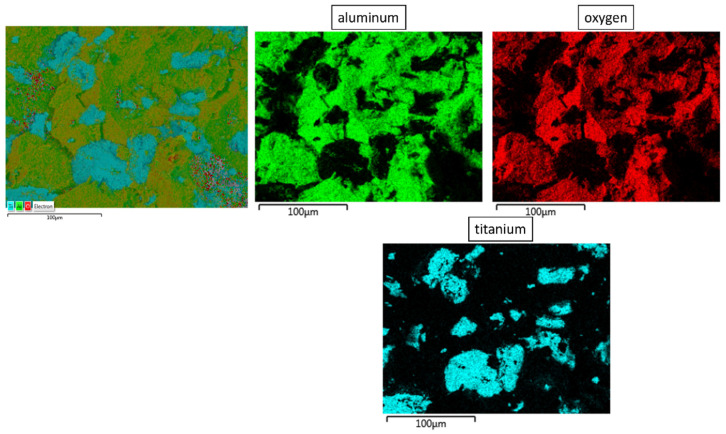
Maps of chemical element distribution on the sample surface.

The SEM image (in backscattered electron mode—BSE) shown in Figure 8 revealed characteristic areas of sintered Al_2_O_3_/Ti composite. In BSE mode, the aluminum oxide phase is presented as dark grey areas, while the titanium phase is presented as light grey. Observations were carried out at the fractured surface. Observation revealed that the titanium particles are uniformly distributed in the alumina matrix. No areas were observed that are over-enriched or deficient in the second phase. The fractured surface is free of secondary cracks, which confirms to a certain extent that the obtained composite does not contain any reported imperfections or structural discontinuities (e.g., large pores). SEM results confirmed that the titanium particles do not form agglomerates in the alumina matrix. In addition, an EDS X-ray spectroscopy investigation was performed to obtain elemental distribution maps of the tested samples. Analysis of the chemical elemental distribution maps, shown in Figure 9, revealed that only aluminum, oxygen and titanium were found. The observation of the whole area of the sample revealed that the titanium agglomerates are evenly distributed in all parts of the composite. The size of the titanium particles equals 20.6 ± 10.1 μm, which corresponds to 27.5% of the initial size and indicates significant fragmentation of the titanium powder during the manufacturing of the composite. A quantitative analysis of the recorded EDS spectra (areas in Figure 8) is presented in Table 4.

XRD phase tests and SEM/EDS analysis showed that after the sintering process, new phases of TiO_2_ and Al_2_TiO_5_ appeared in the formed shapes. Thialite, or aluminum titanate, with the stoichiometry of Al_2_TiO_5_, is a synthetic refractory material that is formed as a result of an equimolar reaction between Al_2_O_3_ and TiO_2_ oxides [35,36,37]. Based on the equilibrium system, it can be concluded that both oxides reacted at 1200 °C, forming the Al_2_TiO_5_ phase. Thialite is characterized by a low coefficient of thermal expansion α (1–2 × 10^−6^/K) [37]. In addition, Al_2_TiO_5_ is characterized by high resistance to thermal shock and relatively high fire resistance (melting point 1860 °C), as well as low thermal conductivity (1–2 W/(m·K)) [37]. What is more, thialite is characterized by low contact angles for liquid non-ferrous metals and high chemical resistance. The special properties of Al_2_TiO_5_ make it possible to use this material and composites; its uses include, among others, use in the automotive industry as DPF particulate filters and/or in the refractory industry as ingots for casting liquid aluminum [38]. Unfortunately, like every material, it has its limitations; in the case of Al_2_TiO_5_, the main factor limiting its use is instability in the temperature range of 750 °C to 1280 °C. At these temperatures, thialite decomposes into the starting oxides Al_2_O_3_ and TiO_2_.

## 4. Summary

The search for new solutions in the field of creating composite materials with unique properties has become the main aim of current research. The aim of the research carried out in this work was to determine the possibility of producing ceramic-metal Al_2_O_3_/Ti composites by slip casting. The main conclusions of this investigation are:

- The suspension used to obtain composites exhibits a non-Newtonian character of flow, with clear thinning under the influence of increasing shear forces. The reason for the drop in suspension viscosity is the decreasing internal resistance under increasing stress, due to the parallel arrangement of fluid components in relation to the direction of the flow.
- The TG curve revealed a two-stage change in the mass of the tested sample. In the first stage, up to a temperature of approx. 600 °C, a weight loss of 0.43% is noted, which is associated with the decomposition of organic additives and water. In the second step, a significant (9.03%) increase in mass is visible in the temperature range of 600–1250 °C. The increase in mass presumably results from the transformations of Ti and TiO_2_, resulting from their oxidation, as well as from the formation of compounds that include Al_2_O_3_, TiO_2_ and Al_2_TiO_5_.
- The sintered composites are characterized by the lack of surface defects such as cracks, microcracks and delamination. The relative density of the fabricated composites was equal to 99%.
- The sintering process resulted in the formation of two new phases: TiO_2_ and Al_2_TiO_5_ (thialite).

## Figures and Tables

**Table 1:** Specification of the powders used in the research based on manufacturers’ data.

Material	Company	Average Particle Size	Density	Purity
α- Al_2_O_3_	Tamei Chemicals Co.	0.100 ± 0.025 µm	3.98 g/cm^3^	99.99%
Ti	GoodFellow Cambridge Limited	75 µm	4.51 g/cm^3^	99.50%

**Table 2:** Theoretical densities of alumina, nickel and titanium oxide powder declared by manufacturers and actual densities measured by the pycnometric method.

Material	Theoretical Density [g/cm^3^] (Declared by the Manufacturer)	Real Density [g/cm^3^] (Pycnometric Method)
α- Al_2_O_3_	3.98	3.997 ± 0.028
Ti	4.51	4.538 ± 0.0162

**Table 3:** Value of dynamic viscosity of prepared ceramic slurries for shear rates of 0.1; 1; 10; and 100 s^−1^.

Series	Viscosity at a Shear Rate of 0.1 s^−1^ (Pa∙s)	Viscosity at a Shear Rate of 1 s^−1^(Pa∙s)	Viscosity at a Shear Rate of 10 s^−1^ (Pa∙s)	Viscosity at a Shear Rate of 100 s^−1^ (Pa∙s)
Al_2_O_3_/Ti (10%)	7.14	1.51	0.37	0.13

**Table 4:** Chemical composition of characteristic areas of the composites. Measurement areas are shown in Figure 9.

Area	Chemical Composition (%wt.)					
	O		Al		Ti	
	%wt.	%at.	%wt.	%at.	%wt.	%at.
1	47.78	60.67	52.22	39.33	-	-
2	-	-	3.27	5.66	96.73	94.34
3	38.26	51.10	61.74	48.90	-	-
4	-	-	4.28	7.35	95.72	92.65

## Data Availability

Not applicable.
